# Human Bocavirus Infection, People’s Republic of China

**DOI:** 10.3201/eid1301.060824

**Published:** 2007-01

**Authors:** Xiao-Wang Qu, Zhao-Jun Duan, Zheng-Yu Qi, Zhi-Ping Xie, Han-Chun Gao, Wen-Pei Liu, Can-Ping Huang, Fu-Wang Peng, Li-Shu Zheng, Yun-De Hou

**Affiliations:** * Chinese Center for Disease Control and Prevention, Beijing, People’s Republic of China; 1 These authors contributed equally to this work.

**Keywords:** human bocavirus, China, children, dispatch

## Abstract

A newly identified parvovirus, human bocavirus (HBoV), was found in 21 (8.3%) of 252 nasopharyngeal aspirates from hospitalized children with lower respiratory tract infection in Hunan Province, People’s Republic of China. Viral loads were 10^4^ to 10^10^ copies/mL. Phylogenetic analysis of the VP1 gene showed a single genetic lineage of HBoV worldwide.

Acute respiratory tract infections (ARTIs) are a leading cause of hospitalization, illness, and death in infants and young children (*1**–**4*). Respiratory syncytial virus (RSV), human metapneumovirus (HMPV), influenza viruses, human coronaviruses, rhinoviruses, and adenoviruses are some of the most important viral agents for this group of patients. However, in a substantial proportion of children with respiratory tract diseases, no pathogen can be identified (*1*).

Until recently, the only parvovirus known to be pathogenic for humans is B19 (*5*). In 2005, a new human virus of the genus *Bocavirus* considered to be pathogenic for humans, provisionally named human bocavirus (HBoV), was described in Sweden (*1*). Subsequently, HBoV infection was reported in children with ARTIs in Australia (*6*), Japan (*7*), Canada (*8*), France (*9*), and the United States (*10*). In our study, 252 nasopharyngeal aspirates (NPA) obtained from November 3, 2005, to April 3, 2006, from hospitalized children with lower respiratory tract infections were analyzed for the presence of HBoV because of associated clinical manifestations.

## The Study

Participants in the study were children <10 years of age who were hospitalized from November 3, 2005, to April 3, 2006, in Hunan Province, People’s Republic of China. They were admitted mostly for bronchitis, pneumonia, and bronchopneumonia; their NPA were collected for investigation of the cause. All children were admitted 2–6 days after the onset of ARTI. All specimens were collected after the parents of the enrolled children had given informed consent.

DNA was extracted from NPA specimens by using the QIAamp Viral DNA Mini Kit (QIAGEN, Beijing, China). HBoV in extracted DNA was detected by PCR amplification of a 291-bp fragment of the NS1 gene as described previously (*6*). To acquire the complete sequence of the VP1 gene, we used primers 5′-GATAACTGACGAGGAAATG-3′ and 5′-GAGACGGTAACACCACTA-3′ based on the published genomic sequence of HBoV (GenBank accession no. NC_007455). The PCR cycle included an initial heating at 95°C for 15 min; 40 cycles of 94°C for 45 s, 50°C for 45 s, and 72°C for 2 min; and a final extension at 72°C for 10 min. Both short and long PCR products were sequenced. Sequencing was performed on an Applied Biosystems 3730 XL DNA Analyzer (Applied Biosystems, Foster City, CA, USA) by using both the forward and reverse primers. The complete sequences of the VP1 gene obtained were aligned with sequences available in GenBank by using Clustal X (ftp://ftp-igbmc.u-strasbg.fr/pub/ClustalX/). A neighbor-joining tree was constructed by the neighbor-joining method using the MEGA 3.1 program (www.megasoftware.net) and sequences of canine minute virus (MVC) and bovine parvovirus (BPV). Human parvovirus B19 (B19) was used as the outgroup.

A TaqMan real-time PCR targeting the NS1 region of HBoV was conducted to quantify the viral load. In brief, 2 μL genomic DNA was amplified in a 25-μL PCR mixture containing 5 μL ABI TaqMan 2× PCR Master mix, 20 μM of each primer, and 20 μM of the probe. The primer sequences used were 5′-TAATGACTGCAGACAACGCCTAG-3′ and 5′-TGTCCCGCCCAAGATACACT-3′, and the probe was 5′-FAM-TTCCACCCAATCCTGGT-MGB-3′. The cycling conditions included initial incubations at 50°C for 2 min and 95°C for 10 min, followed by 40 cycles of 95°C for 15 s and 60°C for 30 s. Plasmid pGEM-T-NS1 containing the target sequences was constructed and used as a positive control for copy number calculation. Sensitivity of the PCR assay was 100 copies per reaction, as determined by dilutions of the plasmid.

RNA was also extracted from each NPA specimen by using the QIAamp Viral RNA Mini Kit (QIAGEN) to screen for HMPV (*11*), RSV (*12*), influenza (A, B, and C) (*13*), parainfluenza (types 1–4) (*13*), and human coronaviruses (229E, OC43, NL63, and HKU1) by standard reverse transcription–PCR technique (*13**–**15*).

HBoV was detected by the diagnostic PCR in 21 (8.3%) of NPA specimens collected from 252 hospitalized children with ARTI. Serum samples available from 2 of the HBoV-positive patients were also positive. Two HBoV-positive patients (patient 7 and patient 10) had coinfection with human coronavirus 229E. Among the HBoV-positive patients, 17 (81%) were male, and 4 (19%) were female (Table). The ages of the infected patients were 2 months to 3 years (median age 10.5 months), with the exception of a 10-year-old boy (patient 8). The most common clinical signs and symptoms were cough (86%), fever (33%), wheezing (33%), and diarrhea (29%) (Table). The 3 main admission diagnoses were pneumonia (6 patients), bronchitis (6 patients), and bronchopneumonia (7 patients). These patients had been admitted to hospital for 2 to 28 days. Chest radiographs were obtained from 12 patients; all showed abnormal findings (6 had airspace shadows, and 6 displayed coarse lung markings). Most HBoV-positive patients had no other underlying illness, with the exception of 1 (patient 3) who had intracranial infection. Although cough and diarrhea were more frequently found in HBoV-infected children (86% and 29%, respectively) than in HBoV-uninfected children (60% and 7.8%, respectively), confirmation of the disease association of HBoV infection requires the analysis of HBoV in a negative control group of healthy children.

**Table:** Clinical data for children with human bocavirus DNA detected in nasopharyngeal aspirate (NPA) specimens*

Patient no.(sex)	Age, mo	Days in hospital	Diagnosis	Signs/symptoms	Chest radiograph	Copies/mL specimen (NPA)
1 (F)	12	5	P	Fever, vomiting, diarrhea	NR	1.2 × 10^10^
2 (M)	18	15	P	Cough, rash	BA-ILM	3.1 × 10^4^
3 (M)	12	28	II	Cough, hypodynamia	RLZ-AS	5.1 × 10^9^
4 (M)	8	3	B	Fever, clonism	NR	4.3 × 10^4^
5 (M)	10	15	BP	Cough	BA-ILM	2.5 × 10^10^
6 (F)	2	2	BP	Dyspnea, cyanosis	RLZ-AS	4.7×10^4^
7 (M)	4	11	BP	Cough, diarrhea	BA-ILM	1.6 × 10^5^ (1.2 × 10^5^)†
8 (M)	120	10	P	Fever, cough	BA-ILM	4.4 × 10^4^
9 (M)	9	19	B	Cough, wheeze	NR	1.8 × 10^9^
10 (M)	2	16	SP	Cough, foaming, ALTE	BAS	6.7 × 10^4^ (4.4 × 10^4^)†
11 (M)	24	7	P	Cough, wheeze	RUZ-AS	1.8 × 10^9^
12 (M)	14	6	B	Cough, wheeze, polypnea	NR	4.2 × 10^4^
13 (M)	36	14	B	Fever, cough, wheeze	NR	4.8 × 10^4^
14 (F)	8	17	B	Fever, cough, wheeze	NR	5.5 × 10^4^
15 (M)	6	8	BP	Cough, polypnea, diarrhea	BA-ILM	2.4 × 10^4^
16 (F)	6	8	FOU	Fever, cough, wheeze	BA-ILM	7.3 × 10^4^
17 (M)	6	5	BP	Cough, diarrhea	BAS	4.5 × 10^4^
18 (M)	15	9	B	Cough, wheeze, diarrhea	NR	6.1 × 10^4^
19 (M)	7	9	BP	Cough, polypnea	NR	3.8 × 10^5^
20 (M)	2	15	BP	Cough, polypnea	BAS	7.6 × 10^4^
21 (M)	9	6	P	Fever, cough, diarrhea	NR	8.3 × 10^4^

*M, male; F, female; P, pneumonia; NR, none reported; BA, bilateral airspace; ILM, increased lung marking; II, intracranial infection; RLZ, right lower zone; B, bronchitis; BP, bronchopneumonia; SP, severe pneumonia; ALTE, apparent life-threatening event; RUZ, right upper zone; AS, airspace shadows; FOU, fever of unknown origin; BAS, bilateral airspace shadows. †Parentheses show virus titer in serum sample.

HBoV viral loads in NPA specimens ranged from 2.4 × 10^4^ to 2.5 × 10^10^ copies/mL (Table). The 2 positive serum specimens (from patients 7 and 10) had 1.2 × 10^5^ and 4.1 × 10^4^ copies/mL, respectively, which were almost equal to those found in their corresponding NPA specimens. Most specimens had HBoV viral loads close to10^4^ copies/mL. However, 5 (24%) NPA specimens had viral loads >10^9^ copies/mL.

The entire VP1 gene of HBoV was sequenced for 5 specimens that had adequate amounts of genomic DNA. An alignment of VP1 sequences obtained from children in China with those previously reported for the prototype strains (ST1 and ST2 strains, GenBank accession nos.DQ000495–DQ000496) showed only minor sequence differences, with a nucleotide identity of 97.7% and an amino acid identity of 98.1%. Thus, HBoV is a highly conserved virus. Phylogenetic analysis of these sequences and those from BPV, MVC, and B19 indicated that HBoV was more related to BPV and MVC (Figure).

**Figure:**
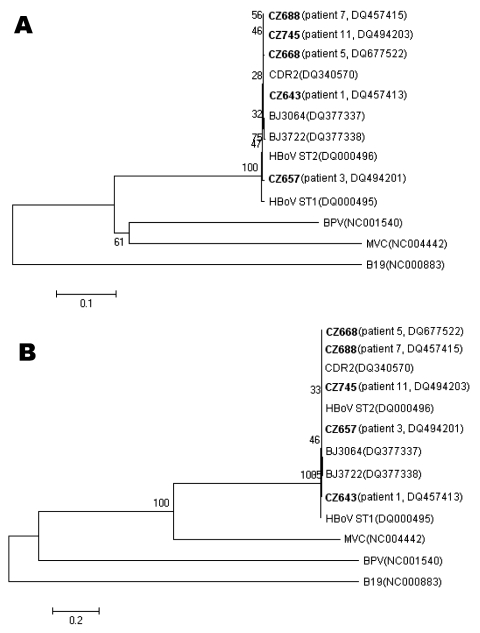
Phylogenetic analysis of the complete VP1 nucleotide (A) and amino acid (B) sequences of human bocavirus (HBoV). Phylogenetic trees were constructed by the neighbor-joining method by using MEGA 3.1 (www.megasoftware.net), and bootstrap values were determined by 1,000 replicates. Viral sequences in **boldface** were generated from the present study, and other reference sequences were obtained from GenBank. Bootstrap values are shown at each branching point. The sequences generated from the present study were deposited in GenBank under accession nos. DQ457413, DQ457415, DQ494201, DQ494203, and DQ677522.

## Conclusions

The prevalence of HBoV in children and the associated illness have not been well characterized. In this study, we found that HBoV was prevalent in infants and young children in China. The 8.3% prevalence rate is higher than rates (3.1%–5.7%) previously reported for children in Sweden, Australia, Japan, Canada, France, and the United States (*1*,*6**–**10*). This difference could be due to the fact that we screened specimens collected during the peak ARTI season. Because this is the first finding of HBoV in children in developing countries, whether the difference also reflects a higher prevalence of the infection in developing countries is unclear.

The symptoms associated with HBoV infection in Chinese children are similar to those reported for children from other countries (*6**–**10*) and are comparable to those observed in children infected with other respiratory viruses, with a predominance of bronchitis or pneumonia (*11**,**13**,**15*). Our results indicate several risk factors for HBoV infection. Consistent with cases reported in the United States (*10*), 57% of our HBoV-positive patients were <12 months of age. Chest radiographs obtained from all 12 patients had abnormal findings. Major diagnoses were pneumonia, bronchitis, and bronchopneumonia. Collectively, these findings support the notion that HBoV infection may be associated with lower respiratory diseases, as suggested by Allander et al. (*1*). We did not find any association between the viral loads and disease severity and could not explain the difference in viral loads among specimens. Nevertheless, the viral loads in serum specimens were similar to those from NPA specimens in the 2 HBoV-positive patients who had both serum and NPA specimens. In our study, 29% of patients had diarrhea, which was also reported in 16% of HBoV–positive patients in the United States (*10*). Unfortunately, we did not collect stool specimens from HBoV-positive patients for viral detection.

Detection of HBoV in serum specimens from 2 patients suggests that HBoV may cause viremia, which was supported by the occurrence of intracranial infection in 1 patient. However, further studies are required to confirm whether HBoV indeed causes viremia. In addition, coinfection with human coronavirus 229E was identified in 2 of the 21 HBoV-positive children in our study. Although RSV, HMPV, and human coronavirus 229E were detected in 13.5%, 7.9%, and 6.0%, respectively, of the patients, no other children were found to be coinfected with HBoV and another virus. This rate of coinfection is lower than that reported for other countries (*1**,**6**,**7**,**10*). Whether seasonal or other factors might account for this difference remains to be determined.

In agreement with previous findings in other countries (*1**,**6**,**7**,**10*), results of our study indicate that HBoV is a conserved virus. Additional epidemiologic studies in different regions and sequence analysis of other genes are required to investigate the overall distribution, seasonality, and genetic variations of HBoV and to examine the origin of current HBoV endemics.
